# Uveal Melanoma

**DOI:** 10.1155/2011/573974

**Published:** 2011-06-30

**Authors:** Vasilios P. Papastefanou, Victoria M. L. Cohen

**Affiliations:** ^1^Ocular Oncology Service, Moorfields Eye Hospital, London EC1V 2PD, UK; ^2^Ocular Oncology Service, St Bartholomew's Hospital, London EC1A 7BE, UK

## Abstract

Uveal melanoma is the most common primary intraocular malignancy and the leading primary intraocular disease which can be fatal in adults. In this paper epidemiologic, pathogenetic, and clinical aspects of uveal melanoma are discussed. Despite the advance in local ocular treatments, there has been no change in patient survival for three decades. Development of metastases affects prognosis significantly. Current survival rates, factors predictive of metastatic potential and metastatic screening algorithms are discussed. Proposed and emerging treatments for uveal melanoma metastases are also overviewed. Current advances in genetics and cytogenetics have provided a significant insight in tumours with high metastatic potential and the molecular mechanisms that underlie their development. Biopsy of those lesions may prove to be important for prognostication and to allow further research into genetic mutations and potential new therapeutic targets in the future.

## 1. Epidemiology

### 1.1. Incidence

Uveal melanoma is the most common primary intraocular malignancy and the leading primary intraocular disease which can be fatal in adults. In the general population it is uncommon with an incidence of 5.3–10.9 cases per million population per years [1, 2]. There has been no change in the incidence of uveal melanoma over the past 30 years in the USA [2]. No change in incidence has been reported for Denmark or Finland. In Sweden, an annual relative decrease of 1% has been reported [3]. The incidence rate in black populations has been shown to be low whether for Africans or African-Americans [1, 4]. The risk is also low among Asian populations [5] and American populations of Asian descent [1]. In Europe a north to south decreasing gradient of melanoma incidence among European populations does support the protective role of pigmentation [6]. Uveal tract melanoma is usually diagnosed in the sixth decade of life with a median age of 55 in most series [7]. The incidence rate has been shown to progressively increase up to the age of 70 years in the USA [2]. In Europe, similar findings have been reported with incidence rates increasing with age, peaking at 75 and then reaching a plateau [6]. 

Most series indicate that both sexes are equally affected with a slight predominance of males [2, 6, 8]. In a review of systemic databases, the age of diagnosis is slightly increased in females (males 59.4, females 61.5) [2]. 

### 1.2. Risk Factors

Development of uveal melanoma has been associated with the presence of ocular or cutaneous melanocytic lesions. Ocular lesions include, primarily, choroidal naevi but also include ocular melanocytosis. The latter manifests as heterochromia, and a dark eye is due to a congenital unilateral hyperpigmentation of the episclera and the uveal tract. The cutaneous conditions associated with uveal melanoma are familial atypical mole and melanoma [9], cutaneous melanoma, and oculodermal melanocytosis (naevus of Ota). 

Another host risk factor is a lightly coloured iris, though there was no definite association found in regards to prior nonocular malignancy or hormonal levels [10]. In regard to environmental factors, weak associations have been made with sun exposure [11]. Some occupations are believed to be associated with an increased risk of ocular melanoma such as arc welders and airline pilots, but this has not been proven. No association has been found with any dietary habits, smoking, or alcohol consumption [9]. 

## 2. Pathogenesis

The development of uveal melanoma has been associated with early oncogenic mutations. These mutations affect pathways involved with the regulation of the cell cycle or the control of cell apoptosis.

### 2.1. Regulation of the Cell Cycle

The retinoblastoma protein inhibits cell cycle progression through the G1-S transition point, and its inactivation by hyperphosphorylation allows cells to reenter the cell cycle [12]. 

These mutations appear to involve the RAF/MEK/ERK pathway. It has been shown in the past that a target of this pathway, *CCND1, *responsible for encoding cyclin D1, is overexpressed in uveal melanomas leading to phosphorylation and inactivation of the retinoblastoma tumour suppressor gene in uveal melanomas [13]. 

An oncogene mutation affecting this pathway is a mutation of the genes *GNAQ* and GNA11 in codon 29. These genes encode GNAQ, the alpha subunit of a heterotrimeric GTP-binding protein that couples G-protein-coupled receptor signaling to the RAF/MEK/ERK and other intracellular pathways. These pathways are important for melanocyte homeostasis. In addition, GNAQ is involved in endothelin signaling which is essential for melanocyte survival early in development [14]. Activation of GNAQ mimics growth factor signaling in the RAF/MEK/ERK pathway leading to transcriptional activation of CCND1. 

*GNAQ *mutation was noted at 49% of the examined uveal melanoma postenucleation samples [13] and 45% of biopsy samples [14, 15], a fact delineating that other mutations could also be responsible. *GNA11 *mutations have been noted at 31.9% of uveal melanoma samples [15]. 

Another molecular event associated with dysfunction of the retinoblastoma protein is the inactivation of the INK4A gene [12].

### 2.2. Control of Cell Apoptosis

The molecular events that have been associated with inhibition of apoptosis in uveal melanoma include inactivation of the p53 pathway [16], defects in the Bcl-2 pathway [17], and activation of the prosurvival PI3K-AKT pathway [18]. 

## 3. Clinical Features

**Figure 1:**
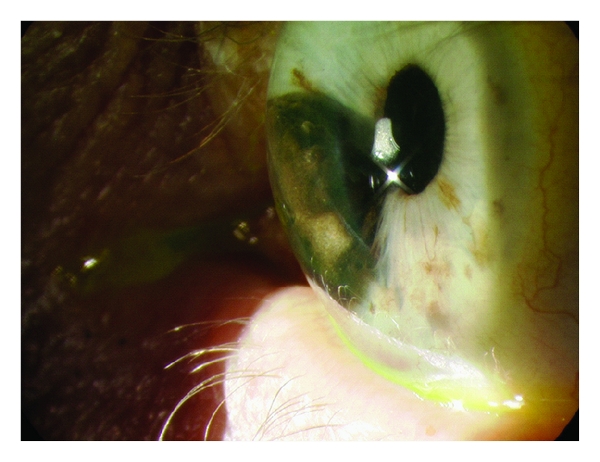
Large iris melanoma occupying a significant percentage of the anterior chamber with associated corectopia.

**Figure 2:**
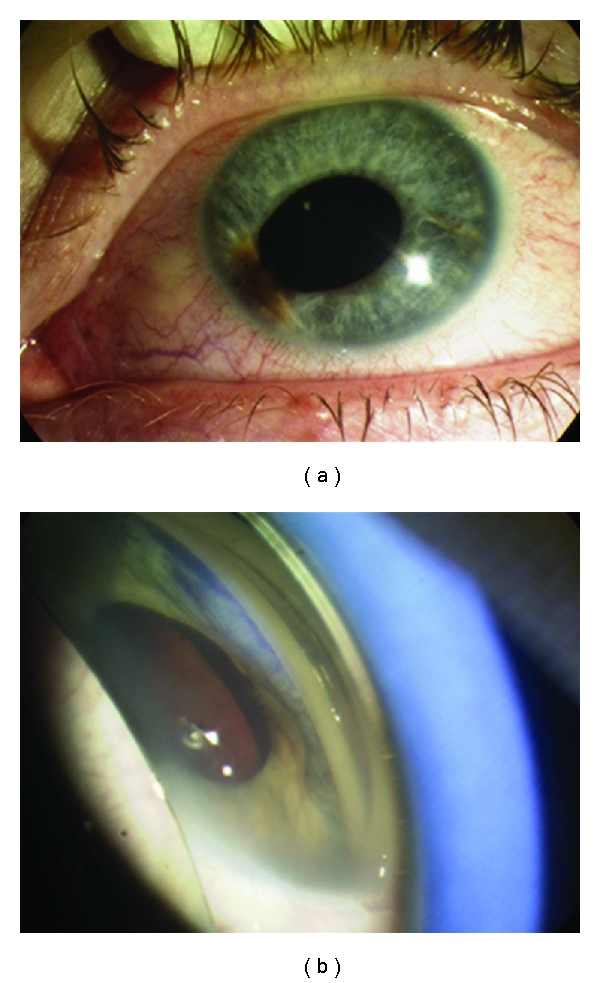
Iris melanoma located in the inferonasal portion of the right eye. Of note the associated corectopia and an episcleral sentinel vessel were adjacent to the lesion. This lesion extended to the anterior chamber and acquired a ring configuration as shown in gonioscopy.

*Iris melanoma* appears as a variably pigmented, usually well-defined mass in the iris stroma and has an 80% predilection to appear in the inferior portion of the iris (Figure 1). Size and shape can also be variable. Less common variables of the iris melanoma are the *diffuse* melanoma, which causes hyperchromic heterochromia of the iris and is associated with infiltration of the trabecular meshwork, *tapioca* melanoma, which has a gelatinous nodular iris appearance, and the *melanoma* of the trabecular meshwork with a ring configuration (Figure 2) [19]. 

**Figure 3:**
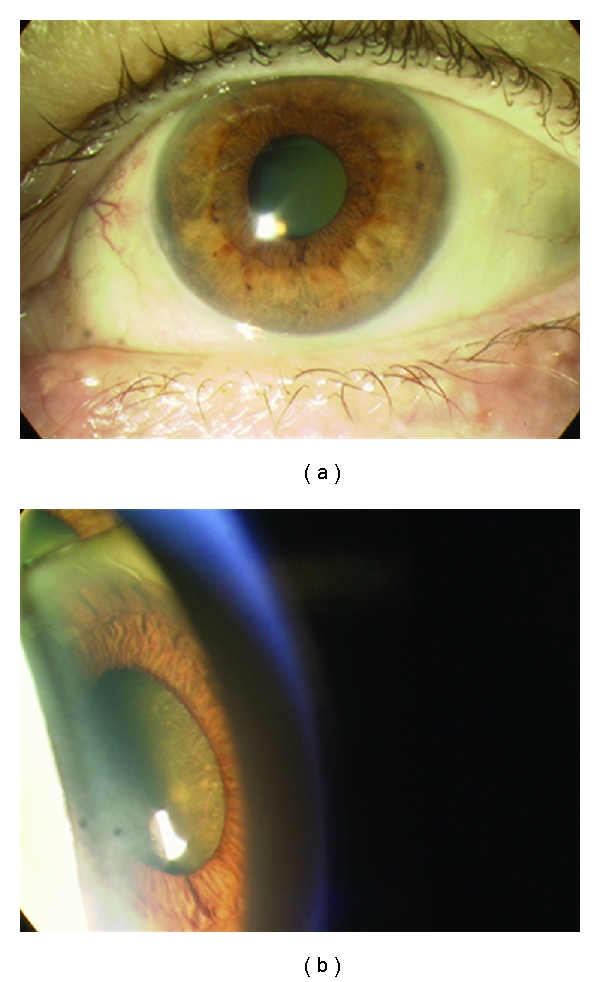
Ciliary body melanoma (a) pigmented area with associated sentinel vessel nasally. (b) gonioscopy reveals the underlying lesion.

**Figure 4:**
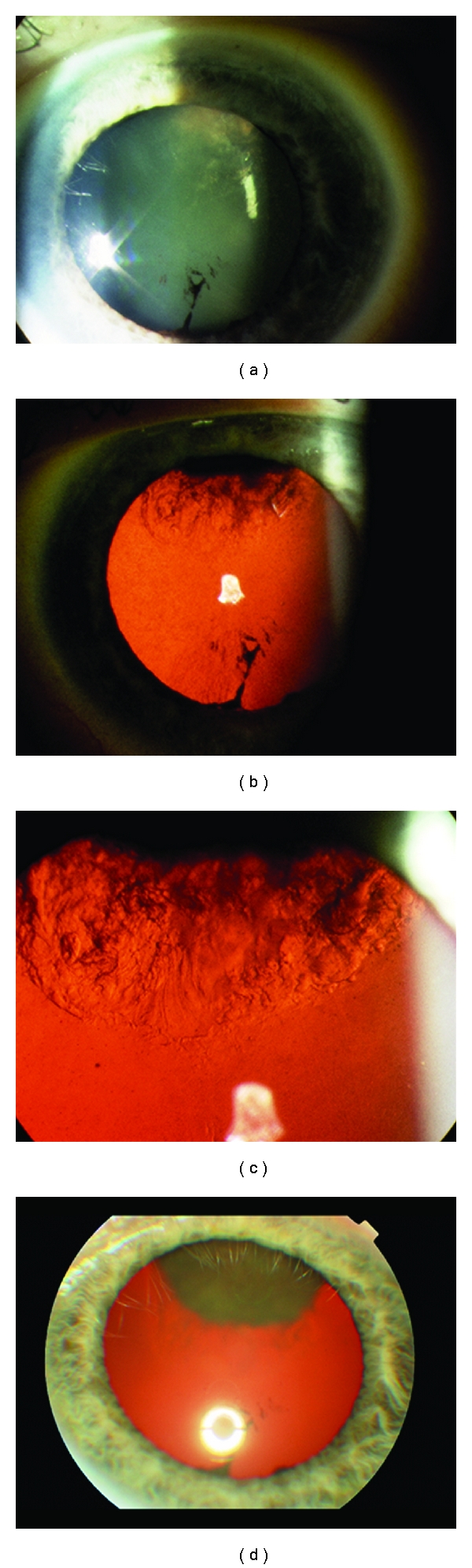
Ciliary body melanoma extending to the lens causing localized cataract.

*Ciliary body melanoma* can attain a larger size before it is recognized clinically. The patient is often asymptomatic. However, a ciliary body mass can cause lens tilting or anterior displacement of the lens which results in an uncorrectable astigmatism. Signs include dilated episcleral vessels (sentinel vessels), dense cataract, and in the case of ring melanoma, raised intraocular pressure. Extrascleral extension is rarely seen at presentation (Figure 3). Usually the lesion has a dome-shaped configuration visualised after dilation of the pupil. Less frequently it can attain a circumferential ring pattern. It can extend toward the lens and cause localized cataract (Figure 4), toward the anterior chamber angle and iris (iridociliary melanoma), or posteriorly into the choroid (ciliochoroidal melanoma) [7]. 

**Figure 5:**
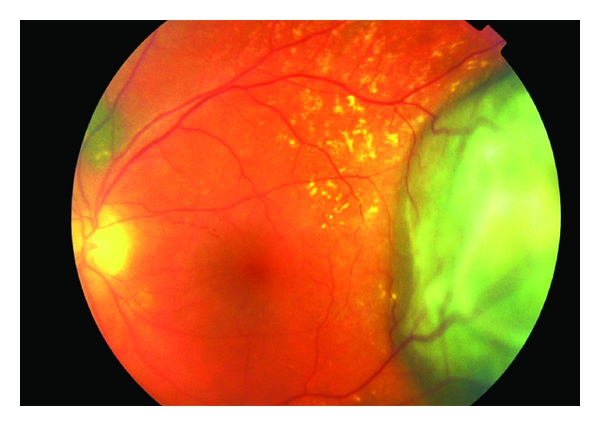
Typical choroidal melanoma with associated nonrhegmatogenous retinal detachment.

**Figure 6:**
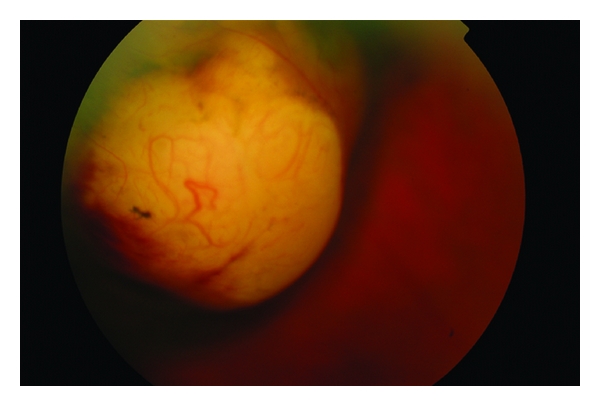
Amelanotic choroidal melanoma. Choroid vessels are visible through the tumor.

*Choroidal melanoma* may present with visual symptoms if it is located at the macula; here it will produce micropsia or visual distortion. Peripheral choroidal melanoma tends to present with a visual field defect or a localised flickering light corresponding to the location of the mass. If an exudative retinal detachment is present, the patient may reach a retinal surgeon first before the diagnosis of melanoma is made. The tumour may be dome or mushroomed shaped. Diffuse choroidal melanoma is a rare aggressive variant. Small choroidal melanomas often have superficial orange pigment known as lipofuscin and associated subretinal fluid (Figure 5). Melanoma is frequently pigmented, and an amelanotic melanoma must be distinguished from other simulating lesions. If the tumour is amelanotic, blood vessels are visible through it and the classical double circulation described with fluorescein angiography may be seen (Figure 6).

## 4. Diagnosis

*Iris melanoma* is usually diagnosed with slit lamp biomicroscopy. However ultrasound biomicroscopy can be used to detect extension of iris melanoma towards the ciliary body and to differentiate it from iris cysts [19]. If there is no evidence of documented growth of the lesion, fine needle aspiration biopsy (FNAB) or conventional iris biopsy is sometimes required to differentiate an iris melanoma from a suspicious iris naevus. 

*Ciliary body and choroidal melanoma* are typically diagnosed with slit lamp biomicroscopy or indirect ophthalmoscopy. The clinical diagnosis can however be supported or confirmed with various ancillary studies.

*Transillumination* is a useful technique to detect ciliary body and anterior choroidal melanomas. It is also used for the delineation of the tumour margins intraoperatively. This technique is performed by placing a fibre-optic point light source on the ocular surface and observing the eye that glows like a light bulb. In most cases the tumour shows up as a dark shadow with well-defined margins. 

### 4.1. Fluorescein Angiography

**Figure 7:**
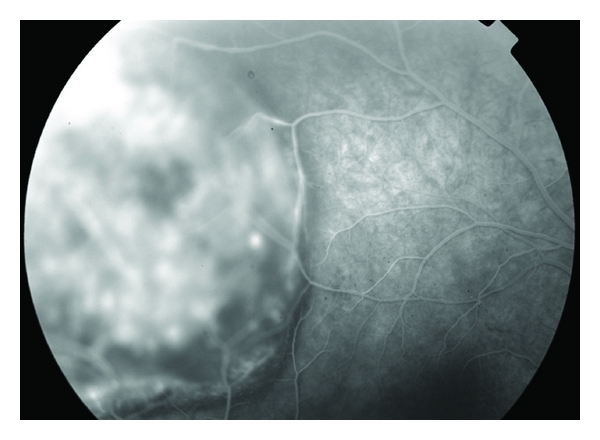
Fluorescein angiogram of a large amelanotic choroidal melanoma, notice the double circulation sign with visible leaking choroidal vessels at the tumor.

Typical findings on the fluorescein angiogram include mottled hyperfluorescence and late staining of the lesion. In the case of an amelanotic melanoma or a large melanoma that has broken through Bruch's membrane, the double circulation sign is noted (Figure 7). In this sign, both retinal and choroidal circulation are visualized.

### 4.2. Ultrasonography

A mode ultrasonography demonstrates medium to low internal reflectivity. However most ocular oncologists use B mode ultrasound which demonstrates the presence of ultrasonographic hollowness and choroidal excavation. Not only is it useful for the examination of a lesion in the presence of a dense cataract or a vitreous hemorrhage but also it can be helpful in measuring the elevation of the tumour. This is important not only in determining the malignant potential of a suspicious lesion but also in determining the response to treatment of a malignant melanoma after radiotherapy or laser treatment [7, 20].

*Computed tomography and magnetic resonance imaging* can be used to determine the presence and degree of extraocular extension, although this is best visualised with orbital ultrasound. *Optical coherence tomography* allows the identification of subretinal fluid associated with a suspicious choroidal naevus [21]. However the clinical importance of fluid on OCT alone is yet to be determined.

### 4.3. Risk Factors of Melanocytic Choroidal Lesions

Eight risk factors for malignant behaviour of melanocytic choroidal lesions have been identified [22]. These include: tumour thickness more than 2 mm at initial diagnosis, presence of associated fluid with the lesion, presence of symptoms, presence of orange pigment on the surface of the lesion, location of the lesion close to the margin of the optic disc (closer than two disc diameters or 3 mm), presence of ultrasonographic hollowness of the lesion at B mode ultrasound, absence of a depigmented halo around the lesion, and absence of drusen. These factors determine the follow-up schedule of the patient or the initiation of treatment [21, 22].

## 5. Treatment

There are many options available for the treatment of uveal tract melanoma. The principal options are enucleation, plaque radiotherapy, proton beam radiotherapy, and transpupillary thermotherapy. 

### 5.1. Enucleation

Enucleation is the traditional method of treating uveal melanomas. It is generally indicated for advanced melanomas that occupy most of the intraocular space or affected eyes with severe secondary glaucoma. It is also indicated for primary tumours that have invaded the optic nerve. Secondary enucleation is indicated if there is definite evidence of recurrence of a tumour initially treated with alternative treatment modalities (see below). Preenucleation radiotherapy involving the use of 2000 cGy of external beam radiotherapy for the affected eye and orbit is no longer advocated as it has not proven to be advantageous over standard enucleation [23].

In the western world enucleation tends to be performed with the placement of an orbital implant onto the socket. There are two major classes of implants: nonporous and porous implants. Nonporous implants are composed of silicone, acrylic or PMMA. The most commonly used porous implants are composed of hydroxyapatite. The porous surface allows fibrovascular growth into the implant, which prevents the extrusion or migration of the implant. Usual sizes are 18–22 mm in diameter.

### 5.2. Plaque Brachytherapy

#### 5.2.1. Rationale

A radioisotope is a typically man-made element with an instable nucleus. The loss of an electron or a neutron is accompanied by the emission of ionising radiation as the radioisotope decays to a more stable element. When a radioactive source is placed in against the sclera or in close proximity to a tumour all structures close to that source are irradiated. Ionizing radiation is absorbed by the exposed tissue resulting in the formation of free radicals, DNA damage, and, eventually, loss of the cell division or cell death.

#### 5.2.2. Technique

Radioactive plaques are typically round curvilinear-shaped episcleral discs of varying diameter. The convex inner surface contains the radioactive source (most commonly ruthenium-106, iodine-125, or palladium-103). The concave external surface consists of a heavy metal (e.g., silver and gold) to shield structures on the outer surface of the plaque. The plaque has two or more eyelets (lugs) to permit suturing to the sclera. Size of the plaque is selected to maintain a 2 mm safety margin around the base of the tumour. Radiation emitted to the apex of the lesion is between 80 and 100 Gy, which is considered to be the effective tumouricidal dose. 

The radioactive plaque is removed 2–7 days after insertion when the calculated dose of radiation has been locally administered.

#### 5.2.3. Complications

**Figure 8:**
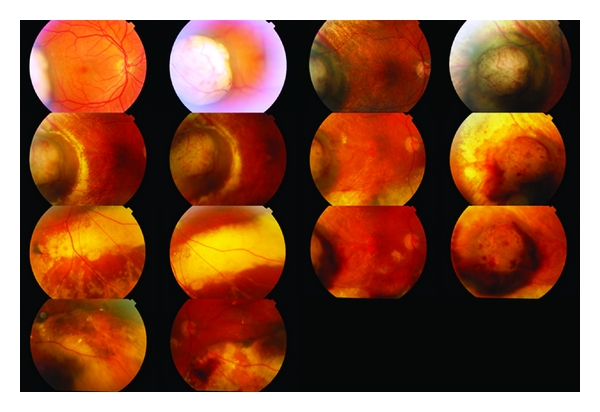
Amelanotic choroidal malignant melanoma treated with plaque brachytherapy. Follow-up images span a period of 7 years. Eventually extensive radiation retinopathy developed.

**Figure 9:**
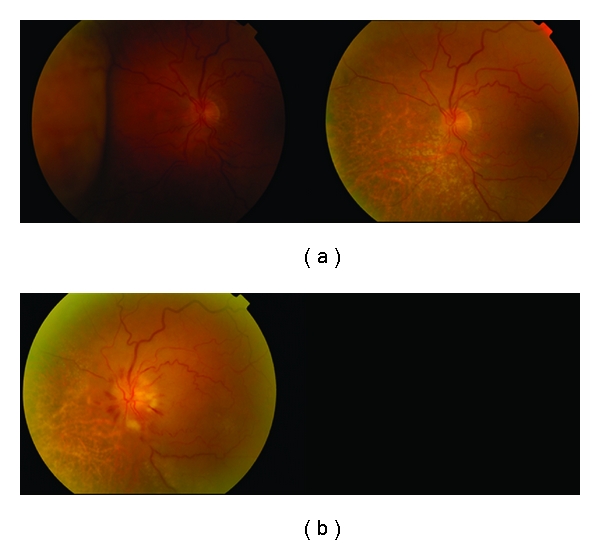
Amelanotic malignant choroidal melanoma treated with plaque brachytherapy. Development of radiation papillopathy and maculopathy one year after treatment.

The complications of plaque brachytherapy include cataract, proliferative radiation retinopathy (Figure 8), radiation papillopathy (Figure 9), maculopathy, neovascular glaucoma [24], and an exudative tumour response [25].

Radiation-induced complications occur on average 18–24 months after plaque treatment [26, 27]. The incidence ranges from 18 to 43% in different series [26, 28]. Clinical signs of radiation maculopathy have been shown to occur to up to 75% of patients in the COMS study [29]. Risk factors for the development of radiation retinopathy include total radiation dose, proximity of the treated lesion to affected structures, diabetes mellitus, and younger age [30, 31]. Panretinal photocoagulation can be used for the treatment of proliferative radiation retinopathy causing regression of neovascularization in 66% of patients in a recent large series [31].

#### 5.2.4. Indications

Plaque brachytherapy is indicated for small choroidal melanomas with evidence of growth, medium-sized uveal melanomas in eyes with useful or salvageable vision, and large melanomas or larger melanomas if in an only eye [32, 33].

#### 5.2.5. Results

The COMS Medium-Sized Tumour Trial has indicated that mortality rates from iodine-125 brachytherapy and enucleation do not differ for up to 12 years after treatment proving the efficacy of radiation in the treatment of uveal melanoma [34]. Survival rates are 82% at 5 years for iodine-125 brachytherapy [25] and 84% for ruthenium-106 [35]. 

The overall tumour recurrence is 10% at 5 years [36]. Treatment failure has been associated with larger tumour size and posterior extension [36, 37]. The secondary enucleation rate is 12–17% at 3–5 years followup [35, 38] and is usually the result of local recurrence or neovascular glaucoma. 

49–55% of patients treated with plaque brachytherapy maintain a best-corrected visual acuity of 6/60 or better and around 30% have 6/18 visual acuity or better in the treated eye [35, 38].

### 5.3. Proton-Beam Radiotherapy

#### 5.3.1. Rationale

Tumour is exposed to a charged proton beam. Charged protons lose their energy in tissue with minimal scatter. The energy deposition occurs at the end of their range (Bragg peak). This property of photons allows a decreased entry chance through normal tissues. The proton beam is conformed to adjust to any tumour size [39].

#### 5.3.2. Technique

Four radiopaque tantalum rings are sutured to the sclera at the border of the lesion to aid with tumour localisation with an ocular X-ray. During the treatment planning, a three-dimensional model of the tumour is superimposed on the normal eye and a face mask and collimator are custom designed for the patient. The fixation angle that will ensure minimal radiation exposure to lens, fovea, and optic disc and maximal exposure to the tumour is determined. Standard treatment is fractionated four times; total dose of 60–70 cobalt Gy equivalents (cGy) is administered.

#### 5.3.3. Indications

Proton bean radiotherapy is indicated for all melanomas and in particular larger melanomas up to 24 mm in diameter and 14 mm in height. Tumours involving the macula, the optic disc, or both are not a contraindication [40]. It is not recommended for very large melanomas that occupy greater than 30% of the ocular volume, or for tumours with extrascleral extension, large retinal detachment, or extensive neovascularisation [41].

#### 5.3.4. Results

**Figure 10:**
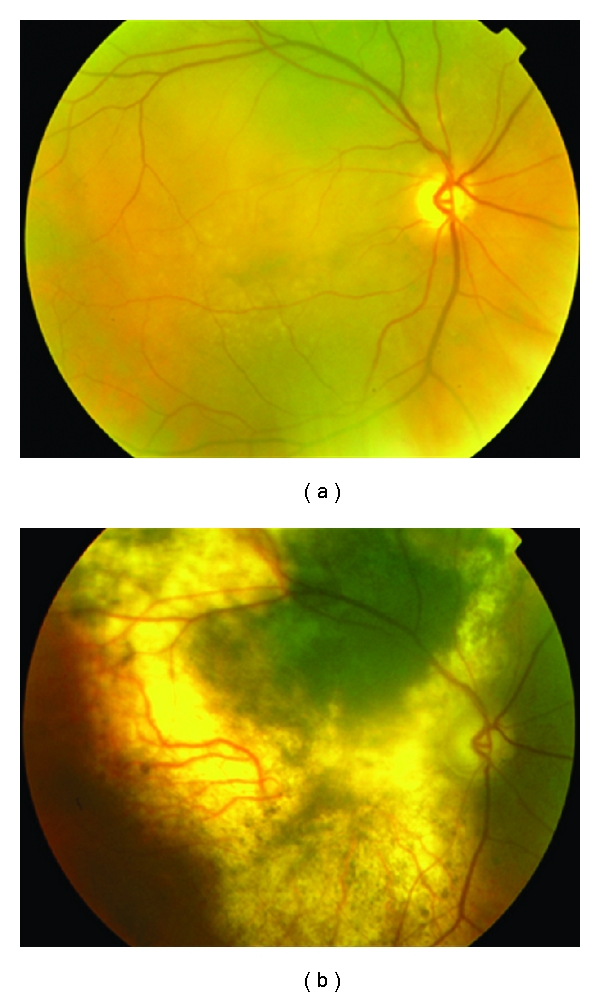
Large choroidal melanoma of the right eye treated with proton beam radiotherapy. Despite extensive atrophy the lesion is flat.

**Figure 11:**
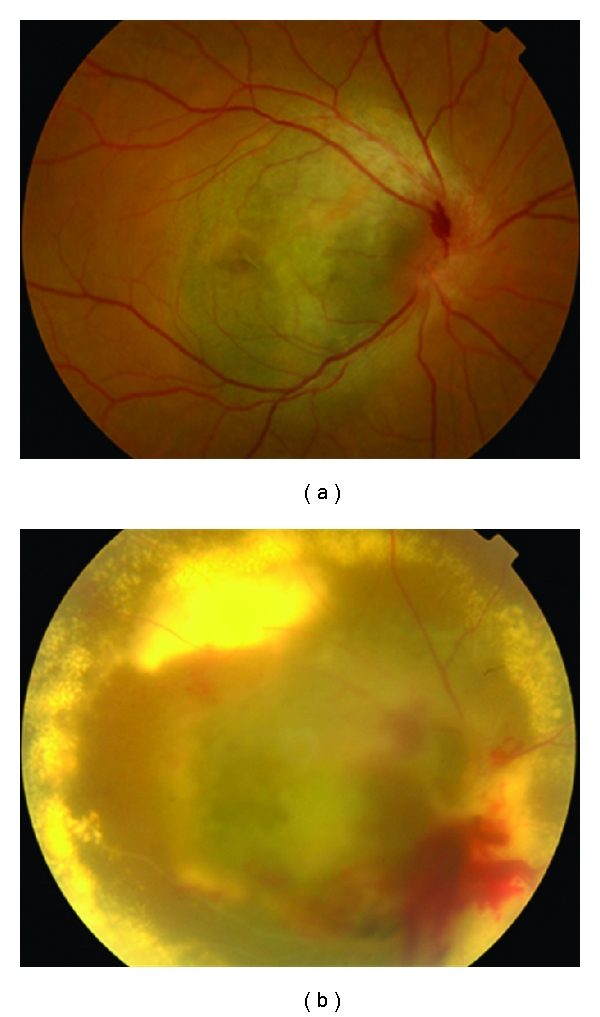
Large juxtapapillary lesion treated with proton beam radiotherapy. Extensive radiation retinopathy and papillopathy developed postoperatively.

Most tumours regress for up to 2 years after treatment. Regression is complete in 15% of patients (Figure 9). Gragoudas et al. have reported vision loss to occur in 68% of patients at 5 years after treatment. This loss has been correlated with proximity of the tumour to the fovea and the optic disc, the elevation and diameter of the tumour, and baseline visual acuity [42]. Two additional risk factors were added in a more recent study [43], namely, diabetes and retinal detachment with percentages fluctuating from 16% for low-risk patients to 99% for high-risk patients. The probability of retaining the eye was 91% at 5 years, 88% at 10 years, and 84% at 15 years after irradiation as indicated by a large recent series [44]. Complications include iris neovascularization [41], posterior subcapsular cataract, radiation maculopathy, and papillopathy (Figures 10 and 11). Survival rates have been shown to be 86% at 5 years, 77% at 10 years, and 73% at 15 years after irradiation. Highest death rates were noted at 3–6 years after treatment [40].

### 5.4. Transpupillary Thermotherapy

Transpupillary thermotherapy (TTT) is a treatment method that utilizes a modified diode laser delivery system to induce hyperthermia to the tumour by delivering heat in the infrared range. Tumour is heated to a temperature of 60–65 degrees [45]. The sensory retina is not damaged as much as in laser photocoagulation. Despite initial results advocating a beneficial effect of TTT [46], high rates of tumour recurrence have been detected in 23–45% of cases [47, 48].

Recurrences have been attributed to the fact that the intrascleral tumour cells do not absorb the emitted heat [49]. Therefore, recurrences have been reported in the orbit due to extrascleral extension. 

Complications of TTT include scotoma, macular traction, vascular occlusion, and hemorrhage [50].

TTT is currently combined with plaque radiotherapy [51] or is applied as secondary treatment to local tumour recurrence after radiotherapy or local resection [52].

## 6. Metastases

### 6.1. Survival Rates

Despite the availability of alternative treatment modalities, the survival rates of patients with uveal tract melanoma have not changed in 30 years. Cumulative rates of metastases in the Collaborative Ocular Melanoma Study at 5 and 10 years after treatment were 25% and 34%, respectively. Common sites of metastases include liver (90%), lung (24%), and bone (16%) [53, 54]. Patients with metastases confined to extrahepatic locations have longer survival (19–28 months) [55]. Median survival for a hepatic metastasis is 6 months with an estimated survival of 15–20% at 1 year and 10% at 2 years, irrespective of treatment [56, 57]. Asymptomatic patients at the time of diagnosis of metastases have a slightly longer survival in relation to symptomatic patients [57]. 

In the case of iris melanoma, distant metastasis to liver or other organs occurs in 5% of patients at 10 years of followup. The risk is higher if the tumour involves the iris root and angle and there is elevated intraocular pressure or extraocular extension [58].

### 6.2. Predictive Factors of Metastatic Potential

#### 6.2.1. Tumour Size

Tumour size is one of the best parameters used to predict metastatic disease. According to the COMS classification, a tumour is defined as small if it measures 3 mm or less in thickness and less than 10 mm in diameter, as medium-sized if 3–5 mm in thickness and 10–15 mm in diameter, and as large if greater than 5 mm in thickness and more than 15 mm in diameter. A comparative analysis of uveal melanoma [59] has indicated that the 5-year survival rates after enucleation were 84% for small, 68% for medium-sized, and 47% for large tumours. Another study [60] indicated that increased tumour thickness increases the risk of metastasis.

#### 6.2.2. Molecular Markers

Dissemination of tumour cells into the blood circulation occurs due to lack of lymphatics in the uveal tract. Haematological markers may, therefore, be useful for the detection of distant metastases. This rationale has prompted the research for determination of potential molecular markers for the early detection of disseminated tumour cells. 

Tyrosinase is an enzyme involved in the synthesis of melanin by melanocytes and melanoma cells. Serum tyrosinase m-RNA levels have been shown to be increased in patients with primary uveal melanoma, and they correlate with metastatic disease. In addition, tyrosinase m-RNA can be used for the indirect quantification of circulating tumour cells and have been correlared with the dimensions of the primary tumour [61]. 

Vascular endothelial growth factor (VEGF) has been proven to overexpress in uveal melanoma cells. It has been suggested that this overexpression is indicative of an angiogenic switch of the uveal melanoma that is associated with a proliferative stage of the tumour and metastatic potential [62]. Overexpression of VEGF originates from abnormal new vessels within the tumour and hypoxia because of the irregular blood flow. VEGF has been traced in uveal melanoma cells and in the aqueous humor in eyes with uveal melanoma [63, 64]. Levels of VEGF have been associated with the metastatic potential of uveal melanoma [65], and serum levels are increased in the presence of micrometastases, and they parallel the extent of liver disease [62, 65]. 

Hepatocyte growth factor (HGF) and its receptor c-Met have been shown to have an important role in the growth of cells in the liver. Increased levels of c-Met in primary tumours have been associated with a high risk of metastatic potential [66]. Activation of HGF by c-Met has been shown to induce increased cell proliferation, downregulate apoptosis, and increase cell motility and invasive ability [67]. 

Insulin-like growth factor-1 (IGF-1) is also produced in the liver, as is HGF. IGF-1 binds to IGF-1R, a surface membrane glycoprotein. Expression of this molecule has been associated with worse prognosis in uveal melanoma [68]. Activation of IGF-1R by binding of circulating IGF-1 increases cell proliferation, prevents apoptosis, and is important for integrin adhesion to the extracellular matrix and invasion of basement membranes. These are essential steps in the formation of metastasis [69]. Therefore when metastatic disease is present, serum IGF-1 levels fall [70]. In a recent study, the coexpression of IGF-1 and c-met in uveal melanoma samples was highly predictive of metastasis [71]. Despite the promising role of serum molecular markers in determining metastatic disease at a subclinical level, their application in metastatic surveillance is limited as there is a wide variability in the normal range within the population. For instance, fluctuations of serum IGF-1 within an individual are more meaningful [70].

#### 6.2.3. Genetic and Cytogenetic Aspects

**Chromosomal Alterations**

Prescher et al. [72] in Germany were the first to describe the chromosome changes seen in uveal tract melanoma which had not been discovered in cutaneous melanoma. The major chromosome alterations have been described in chromosomes 3, 6, 8, and 11. However the most important of these changes is seen in chromosome 3. In short, monosomy 3 (loss of whole of chromosome 3) tends to be found in large uveal tract melanoma in the ciliary body location. These chromosome changes have been strongly linked to patient survival. Monosomy 3 is associated with a 5-year survival of approximately 50%, whereas disomy 3 has been reported to predict 100% survival [73]. In a recent large series of 500 patients with uveal melanoma, those with monosomy 3 had a significantly worse 3-year prognosis in relation to patients with partial monosomy 3 or disomy 3 [74]. Interestingly, these chromosomal abnormalities are significantly correlated with the clinical high risk factors for metastasis in uveal melanoma (such as tumor size at diagnosis and epithelioid cell histology) [75].

Gene expression profiling has been shown to be more predictive of metastatic death than fluorescent in situ hybridization (FISH) analysis of chromosome alterations (12). Using gene expression profiling, melanomas have been categorized into two groups: Class I and Class II. Class I denotes tumours with two copies of chromosome 3 (disomy 3) and other beneficial chromosome changes including gain in chromosome 6p. Class II denoted tumours with only one copy of chromosome 3 (monosomy 3) and other deleterious chromosome changes including gain of chromosome 8p and/ or isochromosome 8p. It is believed that as the tumor undergoes subsequent growth it either gains a fragment of chromosome 6p and becomes a less aggressive Class I melanoma or it loses a copy of chromosome 3 and develops into a Class II melanoma with high metastatic potential. Class II tumors have a greater chromosomal aneuploidy and a significantly different proliferative capacity as indicated by the expression of Ki-67 antigen [73].

This significant discovery has implications on the subsequent management of patients with uveal tract melanoma. For example, patients with Class II tumours are eligible for increased metastatic surveillance and entry into adjuvant treatment trials. At present, the primary surgical management of a uveal tract melanoma remains the same whether the melanoma falls into the category of Class I or Class II.

**Gene Alterations**

Mutations in genes *GNAQ* and *GNA11 *have been associated with the development of uveal melanoma (see Pathogenesis). *GNAQ* and *GNA11* mutations at codon 209 were encountered in 21.7% and 56.5% of metastatic uveal melanoma samples, respectively [15]. In the same series, *GNA11* mutations were more common in locally advanced tumours and in tumours of the ciliochoroidal region. In a recent series of 75 patients [76], *GNAQ* mutations were not associated with disease-free survival despite an occurrence of 53.3%. In addition, no association was found with chromosome status reinforcing the notion that these mutations are an early pathogenetic event and probably are not associated with clinical outcome.

### 6.3. Metastatic Screening

Currently, here is no universally accepted algorithm for metastatic screening in patients with melanoma. In the COMS, chest radiographs and liver function tests were done every 6 months for at least 5 years. Despite high specificity (92%), liver function tests have a sensitivity of less than 15% in the diagnosis of metastatic uveal melanoma [54]. Serum markers indicative of metastatic disease have been shown to be alkaline phosphatase and lactate dehydrogenase [77]. 

Individual case series have demonstrated that whole-body F-18-fluoro-2-deoxyglucose positron emission tomography/computed tomography imaging is a sensitive modality in the followup of uveal melanoma patients [78, 79]. The advantage of this imaging modality is the depiction of metabolic activity as obtained by F-18-fluoro-2-deoxyglucose positron emission tomography with the combination of detailed morphologic characteristics from computed tomography. 

Abdominal ultrasonography is also used for metastatic screening. In a recent study 602 treated patients were screened with biannual abdominal ultrasound. 63 patients developed liver metastases detected by ultrasound. 90% of those patients had metastases in both lobes of the liver, and 70% had more than 10 lesions. One-third of patients with liver metastases underwent complete surgical resection. However, not all metastases could be resected because of the presence of miliary metastases that were not detectable by ultrasound [80]. 

Computed tomography scan has also been used for staging of a malignancy and the detection of metastases. It has been demonstrated that the usage of abdominal CT as a screening tool is often confounded by the presence of benign lesions as cysts, fatty liver, or lesions that are too small in size to characterize [81]. In a recent retrospective study of 198 patients, 55% presented with benign lesions and only 3.3% were found to be metastatic lesions. The likelihood of malignancy increased in relation to the number of lesions detected.

### 6.4. Adjuvant Treatment to Prevent Metastatic Disease

Interferon-alfa-2a has been used as adjuvant treatment after the treatment of melanoma in an effort to prevent the development of metastases as it has been shown to alter the immune response and inhibit cell proliferation. A large recent series [82], however, indicated that the development of metastasis did not differ significantly between patients who received IFN and those who did not [67]. Intra-arterial hepatic fotemustine has shown promising results in the treatment of liver metastases from uveal melanoma (see next section). However, fotemustine did not have a statistically significant survival benefit when used as adjuvant treatment [83]. 

No adjuvant therapy is currently available for ocular melanoma. However, new phase 2 adjuvant treatment trials are underway in Europe for uveal tract melanoma. The London Ocular Oncology Service is collaborating with our European colleagues in Holland in the use of a dendritic cell melanoma vaccination (data not yet published) to prevent the development of metastatic disease in patients at high risk of metastases.

### 6.5. Treatment of Metastasis

A broad spectrum of management options are available for metastatic disease including systemic therapies, direct intra-arterial hepatic administration, and percutaneous hepatic perfusion.

In regard to systemic therapies, the BOLD regimen (bleomycin sulfate, vincristine sulfate (Oncovin), lomustine, and dacarbazine) combined with interferon has shown some response in a small percentage of patients [84].

Fotemustine is an alkylating agent with a high first pass liver extraction leading to hepatic concentrations of 8–47 times higher than in normal tissue. This agent has been evaluated with direct intra-arterial hepatic administration in 101 patients with liver metastases from uveal melanoma and has had promising results with a 36% overall response rate and a median overall survival of 15 months and a 2-year survival rate of 29% [85]. Efficacy of intra-arterial versus intravenous fotemustine is currently being evaluated in a Phase III trial by EORTC (European Organization for Research and Treatment of Cancer).

Another proposed management option is percutaneous hepatic perfusion with melphalan [50]. This has been shown to achieve progression-free disease or stabilisation of patients with uveal melanoma metastases, but unfortunately there was no overall survival benefit [86]. 

#### 6.5.1. Emerging Treatments for Metastases

In light of the molecular events associated with the pathogenesis of uveal melanoma, new therapeutic targets have emerged. Targeting of the effector molecule MEK in the RAF/MEK/ERK pathway with a small-molecule inhibitor, AZD6244, has shown promising results in a small subset of patients with metastatic uveal melanoma doubling the progression-free survival time at 114 days versus 50 days for temozolomide. AZD6244 is currently evaluated in a Phase II randomized trial with temozolomide in patients stratified with GNAQ/11 status [87]. Anti-VEGF treatment is currently under experimental investigation. Bevacizumab has been shown to suppress in vitro growth and in vivo development of micrometastasis of ocular melanoma cells in mice [88]. 

## 7. Conclusion

Uveal melanoma is the most common primary malignancy of the eye affecting approximately 500 patients each year in the UK. Detailed examination and ocular ultrasound invariably allow the clinician to make the diagnosis without the need for a diagnostic biopsy. 

Successful local treatment options, such as plaque brachytherapy and proton beam radiotherapy, allow for the preservation of the eye and vision in some cases. Despite the advance in local ocular treatments, there has been no change in patient survival for three decades. 

Once metastases have developed, prognosis is poor. However, advances in genetics and cytogenetics have helped discover more about the tumours with high metastatic potential and the molecular mechanisms that underlie their development. In that respect, FNAB or conventional biopsy may be important for prognostication and to allow further research into genetic mutations and potential new therapeutic targets.

## References

[1] Scotto J, Fraumeni JF, Lee JAH (1976). Melanomas of the eye and other noncutaneous sites: epidemiologic aspects. *Journal of the National Cancer Institute*.

[2] Singh AD, Topham A (2003). Incidence of uveal melanoma in the United States: 1973–1997. *Ophthalmology*.

[3] Bergman L, Seregard S, Nilsson B, Ringborg U, Lundell G, Ragnarsson-Olding B (2002). Incidence of uveal melanoma in Sweden from 1960 to 1998. *Investigative Ophthalmology and Visual Science*.

[4] Miller B, Abrahams C, Cole GC, Proctor NSF (1981). Ocular malignant melanoma in South African blacks. *British Journal of Ophthalmology*.

[5] Kuo PK, Puliafito CA, Wang KM, Liu HS, Wu BF (1982). Uveal melanoma in China. *International Ophthalmology Clinics*.

[6] Virgili G, Gatta G, Ciccolallo L (2007). Incidence of uveal melanoma in Europe. *Ophthalmology*.

[7] Shields JS, Shields CL, Shields JA, Shields CL ( 1992). Posterior uveal melanoma: clinical and pathologic features. *Intraocular Tumours—A Text and Atlas*.

[8] Frenkel S, Hendler K, Pe’er J (2009). Uveal melanoma in Israel in the last two decades: characterization, treatment and prognosis. *Israel Medical Association Journal*.

[9] Seddon JM, Young TA, Ryan SJ (2006). Epidemiology of uveal melanoma. *The Retina*.

[10] Schmidt-Pokrzywniak A, Jöckel KH, Bornfeld N, Sauerwein W, Stang A (2009). Positive interaction between light iris color and ultraviolet radiation in relation to the risk of uveal melanoma: a case-control study. *Ophthalmology*.

[11] Singh AD, Rennie IG, Seregard S, Giblin M, McKenzie J (2004). Sunlight exposure and pathogenesis of uveal melanoma. *Survey of Ophthalmology*.

[12] Landreville S, Agapova OA, Hartbour JW (2008). Emerging insights into the molecular pathogenesis of uveal melanoma. *Future Oncology*.

[13] Onken MD, Worley LA, Long MD (2008). Oncogenic mutations in GNAQ occur early in uveal melanoma. *Investigative Ophthalmology and Visual Science*.

[14] Van Raamsdonk CD, Bezrookove V, Green G (2009). Frequent somatic mutations of GNAQ in uveal melanoma and blue naevi. *Nature*.

[15] Van Raamsdonk CD, Griewank KG, Crosby MB (2010). Mutations in GNA11 in uveal melanoma. *The New England Journal of Medicine*.

[16] Brantley MA, Harbour JW (2000). Deregulation of the Rb and p53 pathways in uveal melanoma. *American Journal of Pathology*.

[17] Sun Y, Tran BN, Worley LA, Delston RB, Harbour JW (2005). Functional analysis of the p53 pathway in response to ionizing radiation in uveal melanoma. *Investigative Ophthalmology and Visual Science*.

[18] Ehlers JP, Worley L, Onken MD, Harbour JW (2008). Integrative genomic analysis of aneuploidy in uveal melanoma. *Clinical Cancer Research*.

[19] Shields JS, Shields CL (2008). Iris melanoma. *Intraocular Tumours—An Atlas and Textbook 2008*.

[20] Laver NV, McLaughlin ME, Duker JS (2010). Ocular melanoma. *Archives of Pathology and Laboratory Medicine*.

[21] Shields CL, Furuta M, Berman EL (2009). Choroidal nevus transformation into melanoma: analysis of 2514 consecutive cases. *Archives of Ophthalmology*.

[22] Shields CL, Shields JA, Kiratli H, De Potter P, Cater JR, McLean IW (1995). Risk factors for growth and metastasis of small choroidal melanocytic lesions. *Ophthalmology*.

[23] Hawkins BS, Ryan SJ (2006). Schachat AP collaborative ocular melanoma study. *The Retina*.

[24] Seregard S, Damato B, Fleming P, Saunders A (2007). Uveal malignant melanoma: management options—brachytherapy. *Clinical Ophthalmic Oncology*.

[25] Mashayekhi A, Tuncer S, Shields CL, Shields JA (2010). Tumour-lipid exudation after plaque radiotherapy of choroidal melanoma: the role of Bruch’s membrane rupture. *Ophthalmology*.

[26] Quivey JM, Char DH, Phillips TL, Weaver KA, Castro JR, Kroll SM (1993). High intensity 125-iodine plaque treatment of uveal melanoma. *International Journal of Radiation Oncology Biology Physics*.

[27] Quivey JM, Augsburger J, Snelling L, Brady LW (1996). 125I plaque therapy for uveal melanoma: analysis of the impact of time and dose factors on local control. *Cancer*.

[28] Gunduz K, Shields CL, Shields JA, Cater J, Freire JE, Brady LV (1999). Radiation retinopathy following plaque radiotherapy for posterior uveal melanoma. *Archives of Ophthalmology*.

[29] Boldt HC, Melia BM, Liu JC, Reynolds SM (2009). I-125 brachytherapy for choroidal melanoma. Photographic and angiographic abnormalities: the collaborative ocular melanoma study: COMS Report No. 30. *Ophthalmology*.

[30] Gragoudas ES, Li W, Lane AM, Munzenrider J, Egan KM (1987). Risk factors for radiation maculopathy and papillopathy after intraocular irradiation. *Ophthalmology*.

[31] Bianciotto C, Shields CL, Pirondini C, Mashayekhi A, Furuta M, Shields JA (2010). Proliferative radiation retinopathy after plaque radiotherapy for uveal melanoma. *Ophthalmology*.

[32] Robertson DM, Earle JD, Kline RW, Ryan SJ (2006). Brachytherapy for choroidal melanoma. *The Retina*.

[33] Kaiserman N, Kaiserman I, Hendler K, Frenkel S, Pe'er J (2009). Ruthenium-106 plaque brachytherapy for thick posterior uveal melanomas. *British Journal of Ophthalmology*.

[34] Hawkins BS (2001). The COMS randomized trial of iodine 125 brachytherapy for choroidal melanoma, III: initial mortality findings: COMS report no. 18. *Archives of Ophthalmology*.

[35] Bergman L, Nilsson B, Lundell G, Lundell M, Seregard S (2005). Ruthenium brachytherapy for uveal melanoma, 1979–2003: survival and functional outcomes in the swedish population. *Ophthalmology*.

[36] Papageorgiou KI, Cohen V, Bunce C, Kinsella M, Hungerford JL (2011). Predicting local control of choroidal melanomas following 106Ru plaque brachytherapy. *British Journal of Ophthalmology*.

[37] Jampol LM, Moy CS, Murray TG (2002). The COMS randomized trial of iodine 125 brachytherapy for choroidal melanoma: IV. Local treatment failure and enucleation in the first 5 years after brachytherapy. COMS report no. 19. *Ophthalmology*.

[38] Melia BM, Abramson DH, Albert DM (2001). Collaborative Ocular Melanoma Study (COMS) randomized trial of I-125 brachytherapy for medium choroidal melanoma: I. Visual acuity after 3 years, COMS Report No. 16. *Ophthalmology*.

[39] Lane AM, Gragoudas ES, Singh A (2007). Uveal malignant melanoma—proton beam radiotherapy. *Clinical Ophthalmic Oncology*.

[40] Gragoudas ES, Lane AM, Ryan SJ (2006). Charged particle irradiation of uveal melanoma. *The Retina*.

[41] Foss AJ, Whelehan I, Hungerford JL (1997). Predictive factors for the development of rubeosis following proton beam radiotherapy for uveal melanoma. *British Journal of Ophthalmology*.

[42] Gragoudas ES, Li W, Lane AM, Munzenrider J, Egan KM (1987). Risk factors for radiation maculopathy and papillopathy after intraocular irradiation. *Ophthalmology*.

[43] Gragoudas E, Li W, Goitein M, Lane AM, Munzenrider JE, Egan KM (2002). Evidence-based estimates of outcome in patients irradiated for intraocular melanoma. *Archives of Ophthalmology*.

[44] Gragoudas ES, Lane AM (2005). Uveal melanoma: proton beam irradiation. *Ophthalmology Clinics of North America*.

[45] Hanneke JG, Korver J, Korver J, Imhof S, Singh A (2007). Uveal malignant melanoma management options—thermotherapy. *Clinical Ophthalmic Oncology*.

[46] Shields CL, Shields JA, Cater J (1998). Transpupillary thermotherapy for choroidal melanoma: tumor control and visual results in 100 consecutive cases. *Ophthalmology*.

[47] Shields CL, Shields JA, Perez N, Singh AD, Cater J (2002). Primary transpupillary thermotherapy for small choroidal melanoma in 256 consecutive cases: outcomes and limitations. *Ophthalmology*.

[48] Pan Y, Diddie K, Lim JI (2008). Primary transpupillary thermotherapy for small choroidal melanomas. *British Journal of Ophthalmology*.

[49] Journée-de Korver HG, Schalij-Santos NE, Imhof SM (1998). Transpupillary thermotherapy of choroidal melanoma. COMS report No. 6. *American Journal of Ophthalmology*.

[50] Shildkrot Y, Wilson MW (2009). Update on posterior uveal melanoma: treatment of the eye and emerging strategies in the prognosis and treatment of metastatic disease. *Current Opinion in Ophthalmology*.

[51] Keunen JEE, Journe-De Korver GJ, Oosterhuis JA (1999). Transpupillary thermotherapy of choroidal melanoma with or without brachytherapy: a dilemma. *British Journal of Ophthalmology*.

[52] Robertson DM (1999). TTT as rescue treatment for choroidal melanoma not controlled with iodine-125 brachytherapy. *Archives of Ophthalmology*.

[53] Willson JKV, Albert DM, Diener-West M (2001). Assessment of metastatic disease status at death in 435 patients with large choroidal melanoma in the collaborative ocular melanoma study (coms) coms report no. 15. *Archives of Ophthalmology*.

[54] Diener-West M, Reynolds SM, Agugliaro DJ (2004). Screening for metastasis from choroidal melanoma. The Collaborative Ocular Melanoma Study Group Report 23. *Journal of Clinical Oncology*.

[55] Bedician AY (2006). Metastatic uveal melanoma therapy: current options. *International Ophthalmology Clinics*.

[56] Diener-West M, Reynolds SM, Agugliaro DJ (2005). Development of metastatic disease after enrollment in the COMS trials for treatment of choroidal melanoma. Collaborative Ocular Melanoma Study Group Report No. 26. *Archives of Ophthalmology*.

[57] Kim IK, Lane AM, Gragoudas ES (2010). Survival in patients with presymptomatic diagnosis of metastatic uveal melanoma. *Archives of Ophthalmology*.

[58] Shields CL, Shields JA, Materin M, Gershenbaum E, Singh AD, Smith A (2001). Iris melanoma: risk factors for metastasis in 169 consecutive patients. *Ophthalmology*.

[59] Diener-West M, Hawkins BS, Markowitz JA, Schachat AP (1992). A review of mortality from choroidal melanoma: II. A meta-analysis of 5- year mortality rates following enucleation, 1966 through 1988. *Archives of Ophthalmology*.

[60] Shields CL, Furuta M, Thangappan A (2009). Metastasis of uveal melanoma millimeter-by-millimeter in 8033 consecutive eyes. *Archives of Ophthalmology*.

[61] Pinzani P, Mazzini C, Salvianti F (2010). Tyrosinase mRNA levels in the blood of uveal melanoma patients: correlation with the number of circulating tumor cells and tumor progression. *Melanoma Research*.

[62] Filali M, Missotten G, Maat W (2010). Regulation of VEGF-A in uveal melanoma. *Investigative Ophthalmology and Visual Science*.

[63] Missotten G, Notting IC, Schlingemann R (2006). Vascular endothelial growth factor A in eyes with uveal melanoma. *Archives of Ophthalmology*.

[64] Boyd SR, Tan D, Bunce C (2002). Vascular endothelial growth factor is elevated in ocular fluids of eyes harbouring uveal melanoma: identification of a potential therapeutic window. *British Journal of Ophthalmology*.

[65] Sheidow TG, Hooper PL, Crukley C, Young J, Heathcote JG (2000). Expression of vascular endothelial growth factor in uveal melanoma and its correlation with metastasis. *British Journal of Ophthalmology*.

[66] Mallikarjuna K, Pushparaj V, Biswas J, Krishnakumar S (2007). Expression of epidermal growth factor receptor, ezrin, hepatocyte growth factor, and c-Met in uveal melanoma: an immunohistochemical study. *Current Eye Research*.

[67] Peruzzi B, Bottaro DP (2006). Targeting the c-Met signaling pathway in cancer. *Clinical Cancer Research*.

[68] All-Ericsson C, Girnita L, Seregard S, Bartolazzi A, Jager MJ, Larsson O (2002). Insulin-like growth factor-1 receptor in uveal melanoma: a predictor for metastatic disease and a potential therapeutic target. *Investigative Ophthalmology and Visual Science*.

[69] Bakalian S, Marshall JC, Logan P (2008). Molecular pathways mediating liver metastasis in patients with uveal melanoma. *Clinical Cancer Research*.

[70] Frenkel S, Barak V, Zioto O, Pe'er  J Novel biomarkers for detecting uveal melanoma metastases.

[71] Economou MA, All-Ericsson C, Bykov V (2005). Receptors for the liver synthesized growth factors IGF-1 and HGF/SF in uveal melanoma: intercorrelation and prognostic implications. *Investigative Ophthalmology and Visual Science*.

[72] Prescher G, Bornfeld N, Hirche H, Horsthemke B, Jöckel KH, Becher R (1996). Prognostic implications of monosomy 3 in uveal melanoma. *Lancet*.

[73] Onken MD, Worley LA, Harbour JW (2010). Association between gene expression profile, proliferation and metastasis in uveal melanoma. *Current Eye Research*.

[74] Shields CL, Ganguly A, Bianciotto CG, Turaka K, Tavallali A, Shields JA (2011). Prognosis of uveal melanoma in 500 cases using genetic testing of fine-needle aspiration biopsy specimens. *Ophthalmology*.

[75] Damato B, Duke C, Coupland SE (2007). Cytogenetics of uveal melanoma. A 7-year clinical experience. *Ophthalmology*.

[76] Bauer J, Kilic E, Vaarwater J, Bastian BC, Garbe C, De Klein A (2009). Oncogenic GNAQ mutations are not correlated with disease-free survival in uveal melanoma. *British Journal of Cancer*.

[77] Kaiserman I, Amer R, Pe’Er J (2004). Liver function tests in metastatic uveal melanoma. *American Journal of Ophthalmology*.

[78] Freudenberg LS, Schueler AO, Beyer T (2004). Whole-body fluorine-18 fluordeoxyglucose positron emission tomography/computed tomography (FDG-PET/CT) in staging of advanced uveal melanoma. *Survey of Ophthalmology*.

[79] Klingenstein A, Haug AR, Nentwich MM, Tiling R, Schaller UC (2010). Whole-body F-18-fluoro-2-deoxyglucose positron emission tomography/computed tomography imaging in the follow-up of metastatic uveal melanoma. *Melanoma Research*.

[80] Rivoire M, Kodjikian L, Baldo S, Kaemmerlen P, Négrier S, Grange J-D (2005). Treatment of liver metastases from uveal melanoma. *Annals of Surgical Oncology*.

[81] Feinstein EG, Marr BP, Winston CB, Abramson DH (2010). Hepatic abnormalities identified on abdominal computed tomography at diagnosis of uveal melanoma. *Archives of Ophthalmology*.

[82] Lane AM, Egan KM, Harmon D, Holbrook A, Munzenrider JE, Gragoudas ES (2009). Adjuvant interferon therapy for patients with uveal melanoma at high risk of metastasis. *Ophthalmology*.

[83] Voelter V, Schalenbourg A, Pampallona S (2008). Adjuvant intra-arterial hepatic fotemustine for high-risk uveal melanoma patients. *Melanoma Research*.

[84] Bedikian AY, Legha SS, Mavligit G (1995). Treatment of uveal melanoma metastatic to the liver: a review of the M. D. Anderson Cancer Center experience and prognostic factors. *Cancer*.

[85] Peters S, Voelter V, Zografos L (2006). Intra-arterial hepatic fotemustine for the treatment of liver metastases from uveal melanoma: experience in 101 patients. *Annals of Oncology*.

[86] Augsburger JJ, Corrêa ZM, Shaikh AH (2009). Effectiveness of Treatments for Metastatic Uveal Melanoma. *American Journal of Ophthalmology*.

[87] Patel M, Smyth E, Chapman PB (2011). Therapeutic implications of the emerging molecular biology of uveal melanoma. *Clinical Cancer Research*.

[88] Yang H, Jager MJ, Grossniklaus HE (2010). Bevacizumab suppression of establishment of micrometastases in experimental ocular melanoma. *Investigative Ophthalmology &amp; Visual Science*.

